# Three-dimensional image-based high-dose-rate interstitial brachytherapy for mobile tongue cancer

**DOI:** 10.1093/jrr/rrt079

**Published:** 2013-06-03

**Authors:** Ken Yoshida, Tadashi Takenaka, Hironori Akiyama, Hideya Yamazaki, Mineo Yoshida, Koji Masui, Tadayuki Kotsuma, SungJae Baek, Yasuo Uesugi, Taiju Shimbo, Nobuhiko Yoshikawa, Takumi Arika, Yukihiro Koretsune, Yasuo Yoshioka, Yoshifumi Narumi, Eiichi Tanaka

**Affiliations:** 1 Department of Radiology, Osaka Medical College, 2-7, Daigaku-machi, Takatsuki, Osaka 569-8686, Japan; 2 Department of Radiology, National Hospital Organization Osaka National Hospital, 2-1-14, Hoenzaka, Chuo-ku, Osaka city, Osaka 540-0006, Japan; 3 Department of Oral Radiology, Osaka Dental University, 1-5-17 Otemae, Chuo-ku, Osaka-City, Osaka 540-0008, Japan; 4 Department of Radiology, Kyoto Prefectural University of Medicine, 465 Kajiicho, Kawaramachi Hirokoji, Kamigyo-ku, Kyoto 602-8566, Japan; 5 Department of Radiation Oncology, National Hospital Organization Osaka National Hospital, 2-1-14, Hoenzaka, Chuo-ku, Osaka city, Osaka 540-0006, Japan; 6 Department of Oral Surgery, National Hospital Organization Osaka National Hospital, 2-1-14, Hoenzaka, Chuo-ku, Osaka city, Osaka 540-0006, Japan; 7 Institute for Clinical Research, National Hospital Organization Osaka National Hospital, 2-1-14, Hoenzaka, Chuo-ku, Osaka city, Osaka 540-0006, Japan; 8 Department of Radiation Oncology, Osaka University Graduate School of Medicine, 2-2, Yamadaoka, Suita, Osaka 565-0871, Japan

**Keywords:** high-dose-rate interstitial brachytherapy, mobile tongue cancer, image-based plan, dose–volume histogram

## Abstract

To investigate the influence of a 3D image-based treatment-planning method for high-dose-rate interstitial brachytherapy (HDR-ISBT) for mobile tongue cancer, we analyzed dose–volume histogram results for the clinical target volume (CTV) and the mandible. Between October 2010 and November 2011, one and four patients having T2 and T3 tumors, respectively, were treated with HDR-ISBT. Multiplane implantation using 9–15 treatment applicators was performed. Lugol's iodine staining, metal markers, ultrasonography, and magnetic resonance imaging were used to identify the contours of the gross tumor volume (defined as the CTV). The results of the image-based treatment plan were compared with those of the conventional simulated plan on the basis of a reference point 5 mm from the applicator position. The mean D90(CTV) and V100(CTV) were 112% of the prescribed dose (PD) and 98.1%PD, respectively, for the image-based plan, and 113%PD and 97.2%PD, respectively, for the conventional plan. The median CTV_ref_/V_ref_ was 0.23 for the image-based plan and 0.16 for the conventional plan (*P* = 0.01). The mean D_0.1 cm^3^_ (mandible), D_1 cm^3^_ (mandible), and D_2 cm^3^_ (mandible) were 80.1%PD, 62.5%PD, and 55.7%PD, respectively, for the image-based plan, and 109.1%PD (*P* = 0.02), 82.4%PD (*P* = 0.005), and 74%PD (*P* = 0.004), respectively, for the conventional plan). Image-based treatment planning may achieve high-conformity radiotherapy for the CTV and decrease irradiated doses to the mandible.

## INTRODUCTION

Radiotherapy is a standard treatment modality for mobile tongue cancer. Interstitial brachytherapy (ISBT), in particular, shows high local control rates [1–4], equivalent to those of radical surgery. In early-stage disease, partial glossectomy shows good treatment results [5] with less damage to articulatory and swallowing functions. In contrast, for more advanced disease, functional damage caused by tumor resection with reconstruction surgery is controversial because some degree of damage inevitably occurs [6]. Therefore, the Physician Data Query of the National Cancer Institute shows that surgery is the standard treatment for Stage I disease, and radiotherapy is used for T2 and small T3 tongue cancer (http://www.cancer.gov/cancertopics/pdq/adulttreatment).

Low-dose-rate ISBT (LDR-ISBT) has a long history of having an excellent outcome for mobile tongue cancer [1–4]. However, the problems of LDR-ISBT are (i) radiation exposure to medical treatment staff, (ii) difficulty of implant technique for young physicians, and (iii) lack of optimization after implantation. High-dose-rate ISBT (HDR-ISBT) can overcome these problems because there is no radiation exposure to medical staff. Therefore, young physicians can learn the technique without radiation exposure and optimize the dose distribution after implantation. Dose optimization is now more effective with 3-dimensional (3D) image-based planning [7–9] because imaging modalities such as computed tomography (CT) and magnetic resonance imaging (MRI) can precisely show the clinical target volume (CTV) and organs at risk (OARs). These datasets are quite useful for quantitative analysis of dose prescription by dose–volume histogram (DVH), which is objective, shows high reproducibility, and is especially useful for young physicians who wish to study implantation and develop dose-specification skills. There have been some reports of 3D conformal planning for tongue cancer [10–12]. We have also begun to use CT-based 3D treatment planning, using fused MRI images obtained before implantation, so as to examine DVH evaluations for CTV and OARs that could not be performed with two-dimensional (2D) treatment planning. In this study, we present an image-based treatment planning method and DVH analysis to compare our new 3D image-based planning method with a conventional 2D planning method.

## MATERIALS AND METHODS

### Patient and treatment characteristics

Between October 2010 and November 2011, five tongue cancer patients were treated by HDR-ISBT at the National Hospital Organization Osaka National Hospital (Table 1). The median age of the patients was 58 years (range, 47–74 years). All patients had histologically confirmed squamous cell carcinoma. According to the Union for International Cancer Control classification of 2007, one patient was classified with a T2 tumor and four patients were classified with T3 tumors. All tumors were classified as N0. One patient had previously been irradiated with external beam radiotherapy (EBRT) of 60 Gy in 30 fractions to treat cancer of the floor of the mouth. Two patients had severe lung dysfunction and were judged as inoperable. The other three patients refused radical surgery.

**Table 1.** Patient characteristics

Patient	Age	Histology	Stage	Neoadjuvant	Gross tumor volume	Morphological	Irradiation	EBRT	ISBT
number				chemotherapy	(cc)	type	history		
1	47	SCC	T2N0M0	−	3	Indurative	-	-	54 Gy/9 fr.
2	58	SCC	T3N0M0	−	11.2	Infiltrative	+	-	48 Gy/8 fr.
3	74	SCC	T3N0M0	+	11.4	Indurative	-	40 Gy/20 fr.	35 Gy/5 fr.
4	70	SCC	T3N0M0	+	16.4	Indurative	-	-	54 Gy/9 fr.
5	47	SCC	T3N0M0	−	5.9	Infiltrative	-	-	54 Gy/9 fr.

EBRT = external beam radiotherapy, ISBT = interstitial brachytherapy, SCC = squamous cell carcinoma.

Our treatment policy included two treatment protocols as described below. Patients without a previous history of radiotherapy received ISBT as monotherapy at a dose of 54 Gy in nine fractions over 7 days. Three patients were included in this group. Patients with a previous history of radiotherapy received ISBT as monotherapy at a dose of 48 Gy in eight fractions over 7 days. One patient was included in this group. The remaining patient required an exceptional treatment protocol because he had lung dysfunction, and we judged that general anesthesia and implantation in 7 days would be difficult to perform initially. He received EBRT with chemotherapy. However, the tumor response was not very good. Therefore, we switched to ISBT as a boost at a dose of 35 Gy in five fractions over 3 days.

Chemotherapy before irradiation was performed in two patients. One patient received systemic intravenous chemotherapy and the other patient received intra-arterial infusion.

### Applicator implantation

**Fig. 1.**
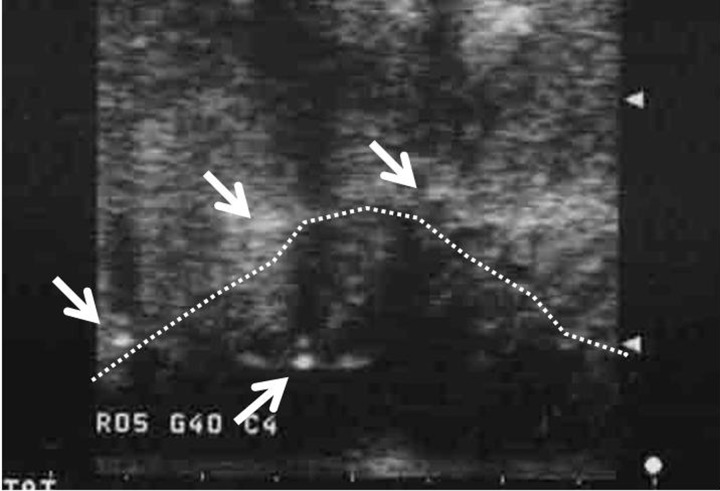
An intraoral ultrasonography image during applicator implantation is shown. Applicators with metal obturators (arrow) and the tumor (dotted line) are clearly visualized.

General anesthesia was used in all patients. Inspection and palpation were performed by at least two physicians, and at least four titanium seed markers were implanted at the anterior, posterior, lateral, and medial edges of the gross tumor volume (GTV) and sometimes at the caudal edge of the GTV. To precisely determine the CTV, we used Lugol's iodine, which is useful for detection of epithelial dysplasia. Implantation was monitored by intraoral ultrasonography (US) using SSD-1000^®^ (Aloka Co. Ltd, Tokyo, Japan) (Fig. 1).

First, we implanted an open-end metal needle (Bevel point needle^®^; Elekta AB, Stockholm, Sweden) in the submandibular region. A custom-made vinyl template on the tongue surface was used to determine the needle exit points. After the needles penetrated the surface of the tongue (medial plane) or the floor of the mouth (lateral plane), flexible treatment applicators were replaced on the needle. Two kinds of flexible applicators were used: one was linearly implanted (Single-leader applicator^®^; Elekta AB) and the other was implanted as a loop (Double-leader applicator^®^; Elekta AB). The loop technique was that used for the applicators implanted near the oropharyngeal region, because buttons on the single-leader applicator on the tongue surface caused irritation.

**Fig. 2.**
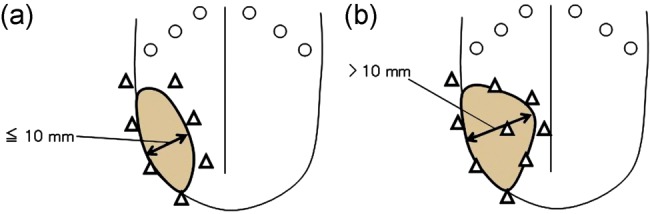
Schemata of implantation are shown. We used two-plane implantation (triangle) when the thickness of the clinical target volume (CTV) (gray color) was ≤10 mm (**a**). If the thickness of the CTV was >10 mm, we used three-plane implantation (**b**).

We always performed implantation with at least two planes (Fig. 2a and b); however, two-plane implantation was used when the GTV thickness was <10 mm (Fig. 2a). Most of the applicators in the lateral plane were inserted by penetration of the mucosa of the floor of the mouth and directly contacted the lateral border of the tongue mucosa. Applicators on the lateral plane were implanted into the lateral edge of the tumor (just outside the GTV). We used scabbard-like silicon tubes for the lateral plane applicators to prevent excessive dose irradiation of the tongue mucosa near the applicators. These scabbard-like silicon tubes were also useful for preventing protrusion of the edematous tongue mucosa between the applicators, which would cause unexpected dose reduction to the GTV. Treatment applicators on the medial plane were implanted as close as possible to the medial side of the GTV. As noted above, if the thickness of GTV was >10 mm, we used three-plane implantation (Fig. 2b). The lateral and medial planes were implanted in the same manner as used for two-plane implantation. The midline plane was implanted between the other planes, and most applicators were implanted into the tumor. We used 9–15 (median, 13) treatment applicators.

### Treatment planning and treatment

All patients underwent CT after implantation, and MRI was used as a reference for CT-based planning to draw the CTV contour. MRI was performed before implantation as a staging modality. We drew the contour of the tongue and mandible on CT and MRI and superimposed using surface-matching or manual registration functions using calculation software Oncentra^®^ brachy (Elekta AB). We used CT, MRI, and metal markers to finally determine the CTV contour.

**Fig. 3.**
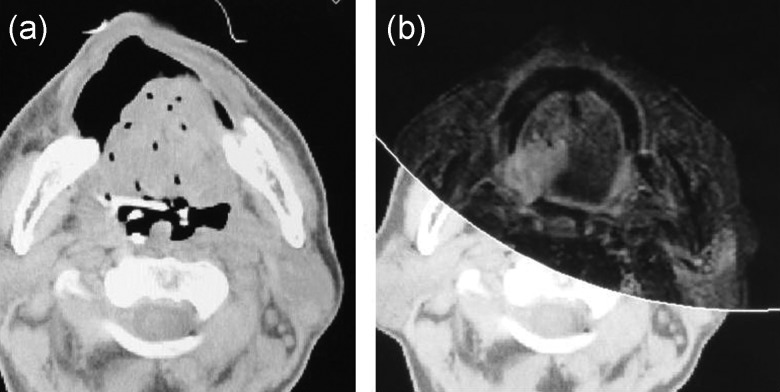
(**a**) A computed tomography image after applicator implantation is shown. Applicator points are clearly visualized in the tongue. However, it is difficult to judge the contour of the gross tumor volume. (**b**) A magnetic resonance image before the applicator implantation was superimposed on the computed tomography image (in the anterior half). It is easier to judge the contour of the gross tumor volume.

We defined the CTV as equivalent to the GTV. The operation records, metal markers, intraoral US, and axial T2-weighted or short-tau inversion recovery MR images were used to delineate the CTV (Fig. 3a and b). The tongue surface extent of the tumor was predominantly evaluated by inspection, with or without Lugol's iodine staining, during implantation as well as by metal markers during treatment planning. We also evaluated the extent of the tumor depth by intraoral US during implantation and by MRI during treatment planning.

We used a modified Paris system with computer optimization (geometrical optimization) and delivered prescribed doses (PDs) to 85% of the basal dose at first. After that, we always modified the isodose shape given in the manual, and sometimes changed the percentage basal dose to adequately cover the CTV by isodose lines of the PD without excessive doses to OARs [13]. Two treatment plans were made for all patients. The first plan was used to treat a wide area for prevention of tumor dissemination [14] and was applied during the first or second treatment fractions. The dwell positions of the treatment source were equivalent to the entire applicator length. The second was a more conformal image-based plan. Because tongue edema may cause worse target coverage, the dwell positions of the treatment source were selected according to the tumor size plus approximately a 10-mm margin.

**Fig. 4.**
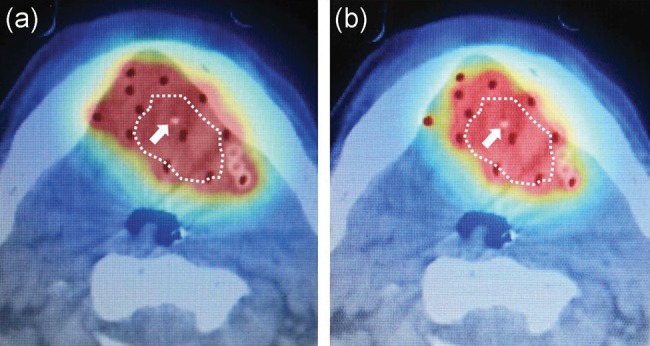
(**a**) A computed tomography (CT) image with dose specification by conventional treatment planning is shown. The contour (dotted line) of the clinical target volume (CTV) was delineated with the aid of a metal marker (arrow), CT, and magnetic resonance imaging. Areas in which more than the prescribed dose was delivered are shown in red. The prescribed dose was delivered over a wider volume than the CTV. (**b**) A computed tomography with dose specification by image-based planning is shown for the same patient. Conformity of the image-based plan was better than that of the conventional plan.

We used an additional treatment planning software (VelocityAI^®^; Velocity Medical Solutions, Atlanta, GA, USA) to sum the two treatment plans and calculate the total DVH values. This software can use a new imaging modality registration method, such as deformable registration, and registration between the imaging modality and dose distribution data of brachytherapy [15]. We imported the CT and MRI datasets and dose distribution curves from the Oncentra^®^ brachy, and the treatment plans were summed (Fig. 4a and b).

A DVH was used to perform dose–volume analysis. The CTV percentage covered by the PD (V100) and the doses that covered 90% [D90(CTV)] or 100% of CTV [D100(CTV)] were calculated. We used a prescribed isodose line (D100(CTV) ≥ PD) to try to cover the CTV. However, we compromised if excessive doses would be delivered to OARs (mandible), or if the hyperdose sleeve was >10 mm. In such cases, our dose specification goal was that the D90(CTV) must be more than the PD. We also analyzed the CTV volume that was covered by the PD (CTV_ref_) and total volume that was covered by the PD (V_ref_). We calculated the CTV_ref_/V_ref_ to compare our image-based plan and the previous conventional 2D treatment plan.

The DVH of the mandible was also calculated. The minimum doses received by the maximally irradiated 0.1 cm^3^, 1 cm^3^, and 2 cm^3^ volumes (D_0.1 cm^3^_, D_1 cm^3^_, D_2 cm^3^_) were analyzed. There was a shortage in this DVH result. We used lead blocks as spacers and shielding devices for the treatment applicator to the gingival mucosa and mandible. In the time-of-planning CT, we used silicon blocks instead of lead blocks to prevent metal artifacts. We could not calculate the influence of lead blocks because Oncentra^®^ brachy has no inhomogeneity correction function. We used microSelectron-HDR^®^ (Elekta AB) with an ^192^Ir source.

### Comparison with the previous conventional treatment plan

To compare our image-based plan with the conventional treatment plan, we created dose distribution curves and DVH data from our 2D conventional planning method that was similar to the Osaka University Hospital method [16]. The dwell positions of the treatment source were fixed at 0–4 cm from the applicator tip, similar to that of the setup for the LDR ^192^Ir pin source. The step size of the treatment source was 2.5 mm, and we used 17 dwell positions for each applicator.

**Fig. 5.**
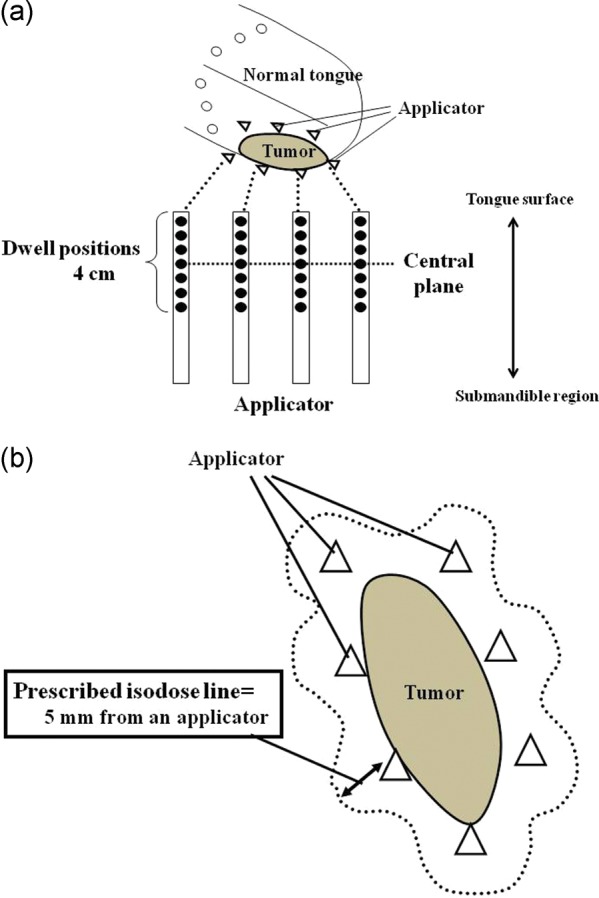
(**a**) The schemata show an axial view of the tongue and applicators (upper part) and a sagittal view of the applicators (lower part). The dwell positions of the treatment source were fixed at 0–4 cm from the applicator tip. The step size of the treatment source was 2.5 mm, and we used 17 dwell positions for each applicator. (**b**) The schema shows an axial view of the tongue and applicators in the central plane. The prescribed dose was delivered to the isodose line that was 5 mm away from the applicator.

Computer optimization was achieved by geometrical optimization of volume without any modification. The reference point was 5 mm from the applicator position in the central plane of the CTV. The PD was delivered to the reference point and the DVH was calculated (Fig. 5a and b).

We compared the DVH results of the image-based plan and the conventional plan, presented as means ± standard deviations. The StatView v5.0 software program was used to perform statistical analyses. Student's *t*-test was used for normally distributed data. The level of statistical significance was set to *P* < 0.05.

## RESULTS

All five patients completed their treatment safely without any complications due to applicator fixation.

The median CTV at the time of HDR-ISBT for each patient was 11.2 cm^3^ (range, 3–16.4 cm^3^). The DVH CTV values are shown in Table 2. The mean D90(CTV) was (112.4 ± 6.1)% of the PD for the image-based plan. The mean D100(CTV) was 86.7% PD ± 19.8% PD. The mean V100(CTV) was 98.1% ± 2.9%. For the conventional plan, the D90(CTV) was 112.5% PD ± 7.1% PD, and the D100(CTV) was 87.2% PD ± 7.9% PD. The mean V100(CTV) was 97.2% ± 2.5%. No significant differences were observed between the two treatment plans. The mean CTV_ref_/V_ref_ was 0.23 ± 0.13 for the image-based plan and 0.16 ± 0.1 for the conventional plan, and the difference was statistically significant (*P* = 0.01).

**Table 2.** Dose–volume histogram values for the clinical target volume

Patient number	D90(CTV) (%prescribed dose)		D100(CTV) (%prescribed dose)		V100(CTV) (%prescribed dose)		CTVref/Vref	
	Image-based	Conventional	Image-based	Conventional	Image-based	Conventional	Image-based	Conventional
1	115	110	103.3	93.3	100	99.1	0.08	0.06
2	116.7	123.3	95	96.7	99.6	99.9	0.20	0.15
3	118.6	115.7	97.1	84.3	99.9	97.3	0.32	0.23
4	106.7	106.7	85	85	97.8	96.1	0.40	0.31
5	105	106.7	53.3	76.7	93.1	93.6	0.13	0.08
Mean ± SD	112.4 ± 6.1	112.5 ± 7.1	86.7 ± 19.8	87.2 ± 7.9	98.1 ± 2.9	97.2 ± 2.5	0.23 ± 0.13	0.16 ± 0.1
	*P* = n.s.		*P* = n.s.		*P* = n.s.		*P* = 0.01	

D90(CTV) = dose that covered 90% of the clinical target volume, D100(CTV) = dose that covered 100% of the clinical target volume, V100(CTV) = clinical target volume percentage covered by the prescribed dose, CTVref = clinical target volume covered by the prescribed dose, Vref = Total volume covered by the prescribed dose.

The DVH mandible values are shown in Table 3. For the image-based plan, the D_0.1 cm^3^_ (mandible), D_1 cm^3^_ (mandible), and D_2 cm^3^_ (mandible) were 80.1%PD ± 19%PD, 62.5%PD ± 12.5%PD, and 55.7%PD ± 11.1%PD, respectively. For the conventional plan, the D_0.__1 cm^3^_ (mandible), D_1 cm^3^_ (mandible), and D_2 cm^3^_ (mandible) were 109.1%PD ± 32.8%PD, 82.4%PD ± 18.3%PD, and 74%PD ± 16.4%PD, respectively. Significant differences were observed between the two treatment plans [*P* = 0.02, 0.005, and 0.004, respectively, for D_0.1__cm^3^_ (mandible), D_1__cm^3^_ (mandible), and D_2__cm^3^_ (mandible)].

**Table 3.** Dose–volume histogram values for mandible

Patient number	D0.1(mandible) (% prescribed dose)		D1(mandible) (% prescribed dose)		D2(mandible) (% prescribed dose)	
	Image-based	Conventional	Image-based	Conventional	Image-based	Conventional
1	85	90	65	75	58.3	70
2	83.3	128.3	70	96.7	63.3	88.3
3	55.7	75.7	44.3	57.1	38.6	48.6
4	106.7	156.7	76.7	103.3	66.7	88.3
5	70	95	56.7	80	51.7	75
Mean ± SD	80.1 ± 19	109.1 ± 32.8	62.5 ± 12.5	82.4 ± 18.3	55.7 ± 11.1	74 ± 16.4
	*P* = 0.02		*P* = 0.005		*P* = 0.004	

D0.1(mandible) = minimum doses received by the maximally irradiated 0.1 cm^3^ volume for mandible, D1(mandible) = minimum doses received by the maximally irradiated 1 cm^3^ volume for mandible, D2(mandible) = minimum doses received by the maximally irradiated 2 cm^3^ volume for mandible.

One patient (#2) had marginal tumor recurrence 7 months after ISBT. The recurrence detected was at the base of the tongue just behind the CTV. The patient died because of local progression 10 months after ISBT. Three patients showed regional lymph node metastasis and underwent radical neck dissection. Four patients were alive without disease when the article was written.

## DISCUSSION

Image-based treatment planning is a more conformal method than conventional needle applicator-based 2D treatment planning. Recently, gynecological brachytherapy has been shifting from 2D planning to 3D image-based planning [7–9] because uterine cervical cancer lesions are well delineated by MRI and CT, and it is possible to draw the 3D contour of OARs (rectum, urethra, and bladder). Because head-and-neck tumors can also be visualized by MRI, and the mandible is well delineated by CT, we initiated image-guided brachytherapy for such tumors. There are only a few studies on image-guided brachytherapy. Nishioka *et al*. used MRI for estimation of tumor volume and shape [10]; however, they did not show the DVH data. Guinot JL *et al*. performed more advanced techniques that used MRI and CT [12]. They used a modified Paris system to calculate the isodoses and delivered their PDs at 90% of the basal dose. After that, they performed a manual adjustment of the isodose curves to decrease the dose to the bone. They did not allow a hot spot joining two tubes to keep the dose non-uniformity ratio under 0.35 (DNR, V150:V100). Neither did they show the DVH data of CTV and OARs.

Image registration between planning CT and MRI before implantation is not the best method because positioning of the tongue is different before and after implantation. However, MRI necessitates relatively longer scanning times and is sometimes difficult in patients just after implantation because of irritability caused by the applicators. In addition, it is difficult to apply MRI in the operating room. To resolve these problems, we used intraoral US for monitoring in the operating room and used MRI performed before implantation to assist CT-based treatment planning in this study. We will try post-implant MRI and MRI-based simulation in a future study to detect the deformed shape of the tongue after implantation. Intraoral US is useful for detection of deep-seated tumors and applicator implant positions, especially to keep the applicator position parallel with the neighboring implant plane [17]. Lugol's iodine staining is useful [18–20] when superficial tumor extension is difficult to detect by MRI, and intraoral US and metal markers are useful for CT visualization of the superficial extension of the GTV [13].

In this study, we used CT with the assistance of metal markers, intraoral US, and MRI to draw the contours of the CTV, which resulted in higher conformity. Although there was no significant difference in the V100(CTV) values, the CTV_ref_/V_ref_ was significantly higher in the conventional plan than in the image-based plan, which means that larger amounts of normal tissue were irradiated in the conventional plan. Because our calculation software had no inhomogeneity correction function and the oral air cavity was included in the V_ref_, our CTV_ref_/V_ref_ absolute values were not perfect. However, we think that the image-based plan is useful when better conformity is needed.

However, there was no difference between the D90/D100(CTV) and V100(CTV) results, which meant that tumor coverage was similar between the treatment plans, although there was a difference in conformity. We think that the reason for this was our precise implantation technique. For example, previously, we often used single-plane implantation for <10-mm-thick tumors. If the tumor thickness was close to 10 mm, we needed to precisely implant in the midline of the tumor to adequately deliver the PD because we adopted the conventional method with a reference point of 5 mm from the applicator. However, if the implant placement was inaccurate or edematous change occurred after implantation, there was a high possibility of poor CTV coverage. We think that the image-based plan would provide better CTV coverage in such cases. In this study, we performed at least two-plane implantation to cover the CTV from the lateral and medial side. This technique has the big advantage of providing a stable dose specification for the CTV. Because of this, we think that our results did not show superior CTV coverage to that of the conventional plan.

We were also able to decrease the irradiated doses to the mandible, although accurate irradiated doses could not be calculated. We used a silicon block at the time of planning, which led to the overestimation of the irradiated doses to the mandible. Our lead block was coated with rubber to prevent unexpected radiation exposure due to scattering radiation; therefore, actual irradiated doses to the mandible must have been lower than the calculated irradiated doses. In future, improvement of dose calculation software will solve this problem. This is the first DVH analysis of brachytherapy for mobile tongue cancer. We plan to investigate the parameters that can allow adequate evaluation of mandible complications.

There are still two problems with image-based HDR-ISBT. One problem concerns target definition. We defined the CTV as equivalent to the GTV; however, Groupe Europeen de Curietherapie–European Society for Therapeutic Radiology and Oncology (GEC–ESTRO) has recommended that the CTV should be equivalent to the GTV plus 5–10 mm [21]. One of the five patients (#2) showed local marginal recurrence in this study, which suggests the CTV criteria should be investigated to determine if a margin should be added to the CTV to give a volume similar to that of the GTV. However, the GTV may change depending on the imaging modality; for example, with or without MRI. We plan to investigate the use of MRI after implantation to draw the GTV more precisely before considering the CTV margin. The second problem is the dose fractionation schedule. We used 6 Gy per fraction, although some reports recommend 3–4 Gy per fraction [12, 21]. We defined a dose fractionation schedule of 60 Gy in 10 fractions initially on the basis of our clinical research [16, 22], but recently started a dose-reduction study that uses 54 Gy in 9 fractions [23, 24]. However, we used conventional treatment planning for our previous research. Because this study showed that the image-based plan gave results different from those of the conventional plan, we need to re-evaluate this problem. The DVH results of the image-based plan were better than those of the conventional plan. Thus we expect that gingival and mandibular complications will decrease without compromise of local control using the image-based plan. Further investigation is necessary to define adequate target definition and a dose fractionation schedule.

## CONCLUSION

In summary, we used intraoral US, metal markers, CT and MRI to investigate image-based brachytherapy for mobile tongue cancer. Our findings showed that the technique may decrease irradiated doses to the mandible without compromising CTV coverage.
